# Measuring corneal hysteresis: threshold estimation of the waveform score from the Ocular Response Analyzer

**DOI:** 10.1007/s00417-012-2053-1

**Published:** 2012-05-09

**Authors:** Marcelo Ayala, Enping Chen

**Affiliations:** Glaucoma Department, Karolinska Institutet, St. Erik Eye Hospital, Polhemsgatan 50, SE-112 82 Stockholm, Sweden

**Keywords:** Waveform score, Ocular response analyzer, Threshold

## Abstract

**Background:**

The aim of this study was to determine a threshold waveform score (WS) for the best score value (BSV) in the Ocular Response Analyzer (ORA).

**Methods:**

Retrospective study. One hundred and thirty-three healthy adults were recruited. Measurements were done with the ORA 2.04.

**Results:**

Two hundred and sixty-six eyes were analyzed. Mean age was 56.49 ± 15.97 years. The mean waveform score of the BSV was 7.39 ± 1.32. The waveform scores ranged from 2.8 to 9.7. Kolmogorov–Smirnov test for normality was significant (*p* ≤ 0.0001).

Linear regression showed a significant positive correlation between IOPg (measured with the ORA) and IOP measured with Goldmann applanation tonometry (*p* ≤ 0.0001), as well as significant negative correlation between the difference IOPg–IOP Goldmann and waveform score of the BSV values. Threshold estimation considering 95 % confidence interval was 7.23. Meanwhile, threshold estimation considering the difference IOPg–IOP Goldmann, for 3 mmHg, was 6.7.

**Conclusions:**

When using the ORA device, we recommend that clinicians try to obtain several waveform score measurements of 7 or above. Waveform scores lower than 7 may render less reliable results.

## Introduction

The ocular response analyzer (ORA) is a device that measures corneal biomechanical properties. The device was developed by Reichert Ophthalmic Instruments (Reichert Inc., Buffalo, NY, USA). Recent studies have shown that corneal biomechanical factors may help in the understanding of additional risk factors for glaucoma [1–4]. ORA is a non-invasive device that utilizes a rapid air impulse to apply force to the cornea. An air pulse causes the cornea to move inwards. Milliseconds after applanation, the air pump shuts off and the pressure declines. As the pressure decreases, the cornea begins to return to its normal configuration. The applanation detection system monitors the cornea and two independent pressure values are derived from the inward and outward applanation events. The average of these two pressure values provides a repeatable, Goldmann-correlated IOP value (IOPg). The difference between these two pressure values is the corneal hysteresis (CH).

Corneal hysteresis (CH) is a measurement of corneal resistance to deformation. This measurement in turn indicates the viscoelastic properties of the cornea. Different materials have different viscoelasticity depending on the ability they have to bounce back to their original state after having been deformed. Low CH demonstrates that the cornea is less capable of absorbing (damping) energy. It has been suggested that increased damping capability may permit an eye to more effectively buffer potentially harmful IOP fluctuations. In theory, this improved buffering might result in reduced stress on both the optic nerve and peripapillary scleral tissues. CH has shown to vary between individuals. Sullivan-Mee et al. [5] found mean CH in normal individuals to be 9.7 mmHg, and Luce et al. [6] found 9.6 mmHg; meanwhile, Ayala M found a value of 9.8 mmHg among normal individuals [4].

CH measurement also provides a basis for two additional new parameters: corneal-compensated intraocular pressure (IOPcc) and corneal resistance factor (CRF). IOPcc is a pressure measurement that utilizes the new information provided by the CH measurements to provide an IOP value that is less affected by corneal properties. CRF appears to be an indicator of the overall “resistance” of the cornea, and is a measurement of the cumulative effects of both the viscous and the elastic resistance encountered by the air jet while deforming the cornea.

A graphic representation of the corneal response after each measurement is displayed in the ORA machine. A typical applanation–pressure plot shows two applanation peaks corresponding to the inward and outward applanation. It depends on the experience of the examiner on reading the ORA waveform. The ORA version 2.04 has incorporated an index called Waveform Score on a scale of 0 to 10. The higher the number, the more reliable the measurement data will be. It is recommended to take several measurements and delete the unreliable ones. Among multiple measurements (up to four measurements can be stored) the signal with the highest Waveform Score (WS) is highlighted as the best score value (BSV). Lam et al. [7], examining 64 normal Chinese adults, recommended taking three measurements, with all the signals having a WS of 3.5 or above. However, Ehrlich et al. [8] in a recently published study used a WS of 6.5 as a cut-off score. Similar results were described by Mandalos et al. [9], who described a higher inter-observer reproducibility when using a cut-off value for WS of 6.0.

The aim of this study was to determine a threshold value for the BSV measurements.

## Materials and methods

This was a retrospective study. One hundred and thirty-three adults were recruited at the St. Erik Eye Hospital in Stockholm. Both eyes were included.

All included subjects were healthy adults. Included subjects were relatives of patients or personnel working at the hospital. They underwent an ophthalmological examination that determined that they did not suffer from ocular diseases. A comprehensive medical and ocular history was obtained. Ophthalmological examination was performed before including patients in the study. Visual acuity, IOP measurements, slit-amp examination and optic nerve status were registered. Visual acuity was recorded using a Snellen chart; all included patients has a visual acuity of 1.0–0.9 with or without glasses. No subject showed astigmatism of more than ± 1 diopter. IOP was measured using a Goldmann applanation tonometer on the same day and after the ORA measurements were registered. All ORA measurements were performed by an assistant nurse; meanwhile, Goldmann applanation measurements were performed by an ophthalmologist (MA). The results were blinded. Slit-lamp examination of the anterior chamber was performed using a Topcon slit lamp. Then, the optic nerve status was evaluated using a 90-D lens through an undilated pupil.

The cular response analyzer (ORA 2.04) was used to perform the measurements.

Four measurements were performed, and the best score value (BSV) was chosen for analysis.

The authors adhere to the tenets of the Declaration of Helsinki.

### Statistical analysis

The Kolmogorov–Smironov test was used to check for normality of the ORA BSV measurements. The 95 % confidence interval for the mean BSV values was calculated. Linear regression analysis was performed to test correlation between IOPg (dependent variable) and IOP measured with Goldmann tonometer. For threshold estimation, two different techniques were used: 1) calculation of 95 % confidence interval of the waveform score from all BSV measurements in all the patients included, and 2) regression analysis of “difference measured in mmHg between IOPg (dependent variable) and IOP Goldmann” and the waveform score. All statistical analysis was performed with the STATA statistical software (Statacorp,4905 Lakeway Drive, College Station, TX, USA).

## Results

Two hundred and sixty-six eyes (133 subjects) were analyzed. There were 63 men and 71 women. Their mean age was 56.49 ± 15.97 years.

**Fig. 1:**
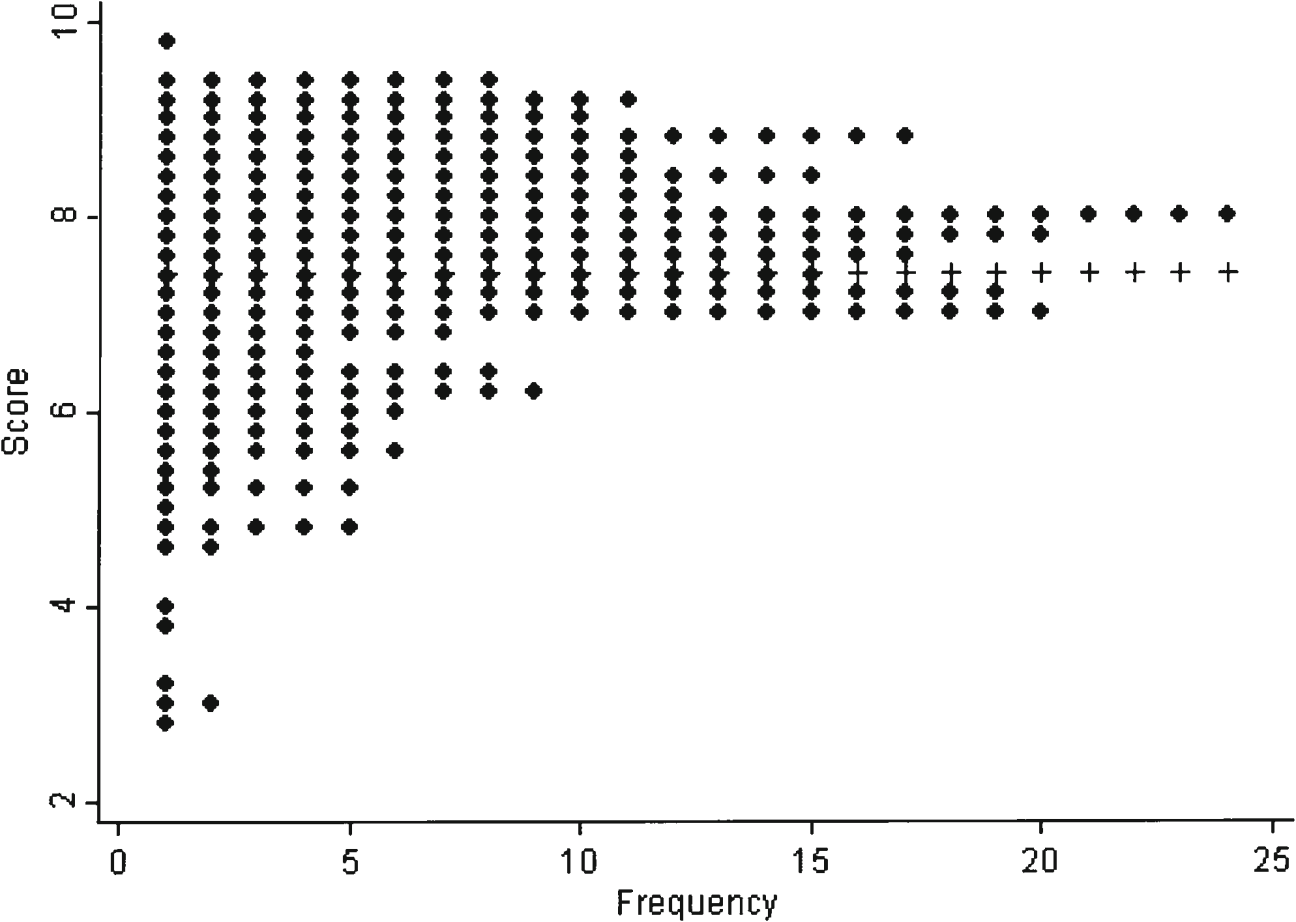
Distribution of the waveform of the BSV measurements

The mean waveform score of the BSV was 7.39 ± 1.32. The waveform scores ranged from 2.8 to 9.7. The Kolmogorov–Smirnov test for normality was significant (*p* ≤ 0.0001). Distribution is shown in Fig. 1. The 95 % confidence interval for the mean waveform score of BSV measurements was 0.16 (7.23–7.55).

**Fig. 2:**
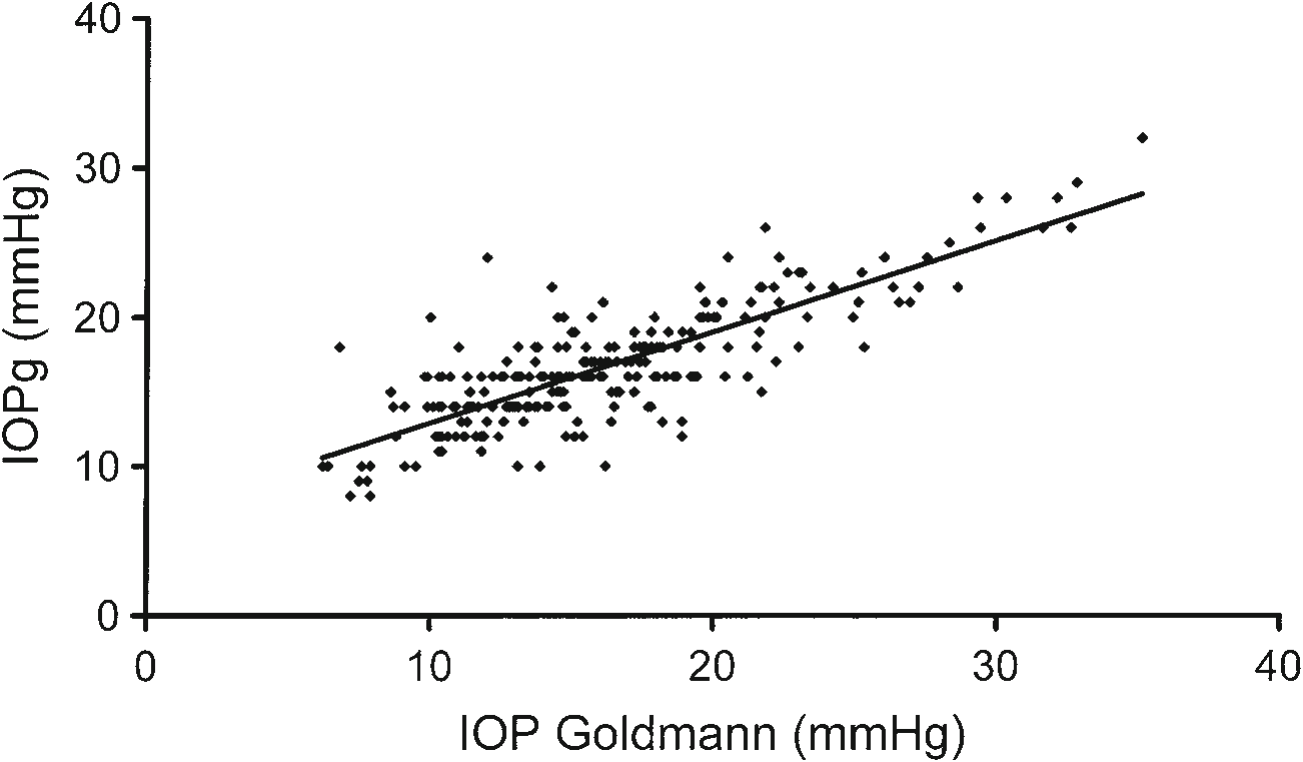
Regression analysis showing a significant positive correlation between IOPg (measured by ORA) and IOP measured by the Goldmann tonometer

**Fig. 3:**
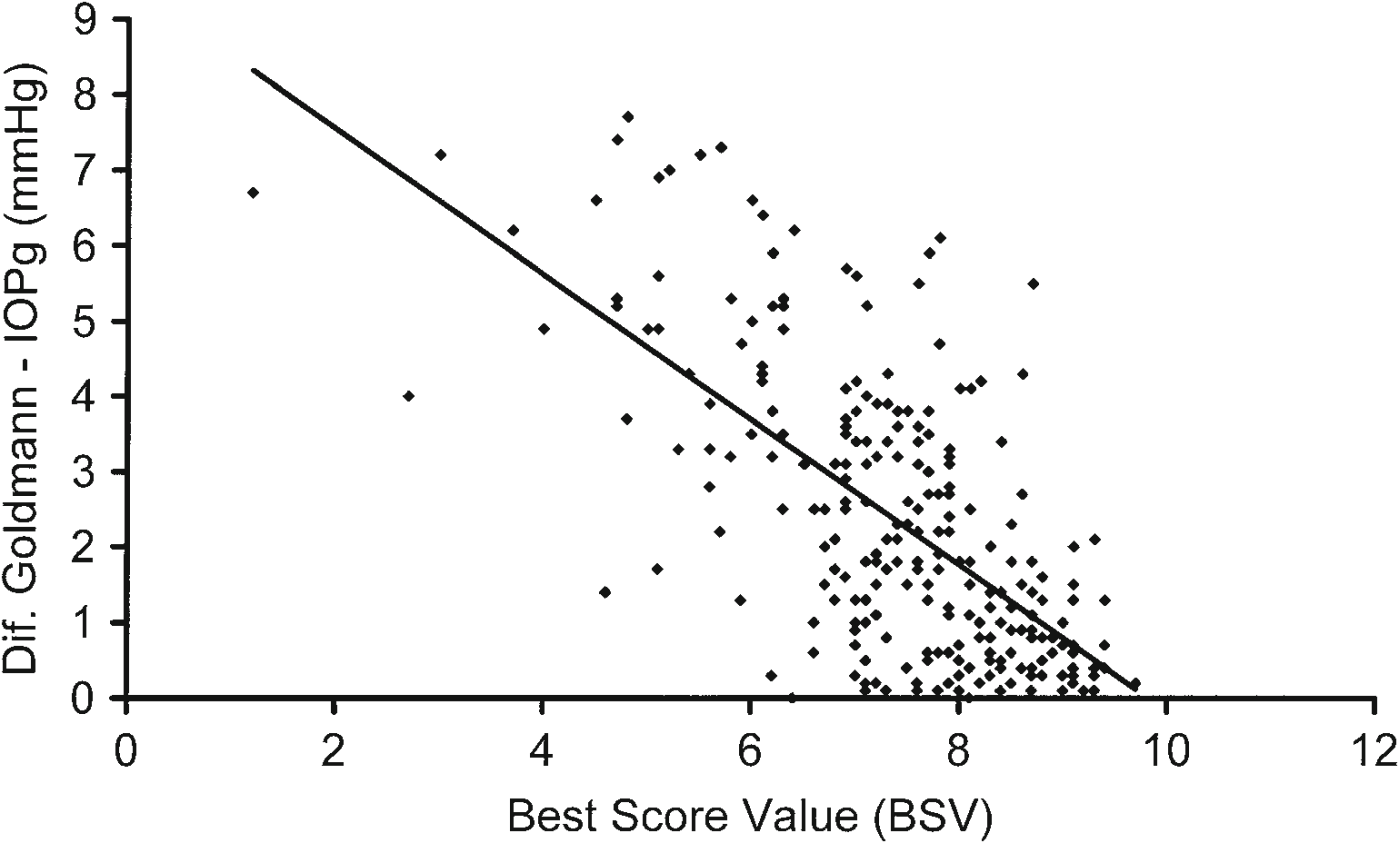
Regression analysis showing a significant negative correlation between the difference in IOP measured by Goldmann and ORA and the waveform score of the BVS measurements. The difference in IOP increases as the waveform score (reliability) decreases

Regression analysis for IOP measured by ORA (IOPg) and IOP measured by Goldmann applanation showed a significant positive trend, increased IOPg with increased IOP Goldmann (*p* ≤ 0.0001) (see Fig. 2). Regression analysis for the difference between IOP measured with ORA (IOPg) and the IOP measured with Goldman applanation versus the waveform score of the BSV showed a significant negative trend, increased difference between the ORA and the Goldmann measurements with decreased waveform score (*p* ≤ 0.0001). See Fig. 3.

## Discussion

The Reichert ORA provides us with new measurements helping us to understand the biomechanics of the cornea. As ORA has been developed, it has been uncertain how the derived values should be used in clinical practice. In the newest version of the Ocular Response Analyzer, a new component (Waveform Score) was added to show the quality of the measurement. The manufacturer has no recommendations with regard to a cut-off level for the Waveform Score (WS), and only one study has been conducted concerning this issue [7]. However, the recommended WS value might not be reliable, as it has not yet been confirmed by any other study. Several studies have taken three ORA measurements and derived a mean value. This is also recommended by Lam et al. [7], even though they did not find a significant difference when comparing the mean value of four measurements to the best signal value (BSV).

According to the manufacturer, a new set of 37 waveform-derived different parameters has been included to better differentiate the signal shape (waveform) of each cornea. The Waveform Score is derived from five of the parameters that according to the manufacturer best described the quality of the measurements. Among included parameters are the areas, heights, and width of the peaks. The areas of the peaks are measured at the intersection between the peak-lines and the air-lines. The heights are evaluated as the difference between the highest and the lowest point in the peaks. The widths are evaluated at the base of the peaks. Another parameter evaluated is the ratio between height and width. The slopes of the peaks are also considered, both the rate of increase and the rate of decrease from the peak to the baseline. The manufacturer recommended to make several measurements and chose the highest Waveform Score (WS) or what is called the best score values (BSV). The results might be different if an average of values is calculated. In cases where an average WS is calculated, then an average value of all the other measurements (like CH, CRF, IOPg, IOPcc) must also be calculated. All these average values are not provided by the ORA device, and in this case they must be calculated manually.

The aim of this study was to estimate a threshold for acceptance of BSV. The higher the waveform score, the higher the reliability of the measurements will be. If we consider threshold estimation based in the normal population distribution included in the study, and the lower 95 % confidence interval for the mean of the BSV as the cut-off value, a signal scored <7.23 should be discharged. In our study, IOP measured with the ORA device (IOPg) correlated well with the IOP measured with the Goldmann applanation tonometer. This correlation decreased when the ORA measurements were not so reliable; with decreased BSV, an increased difference between the two methods for IOP measurement was found. These results agree with a previous study by Erlich et al. [8], who described that an increased WS could predict a smaller difference between IOP measured by Goldmann tonometry and IOPg (ORA). If we considered threshold estimation based on the difference in mmHg between the ORA measurements and the IOP measured with the Goldmann applanation, with a difference of 3 mmHg, the cut-off value for BSV would be 6.7. The BSV is a continuous variable, and to set a cut-off value is more difficult than when a dichotomous variable is examined.

Our results contrast with the results obtained by Lam et al. [7], who estimated a value of 3.50 and signals under this level might be discharged. Difference among the studies can be attributed to a different population studied. Subjects included by Lam et al. [7] were younger (26.3 ± 6.8 years) than subjects included in our study (56.49 ± 15.97 years). Subjects included in our study resemble closer to our usual patients with regard to age distribution. Other possible explanation for the difference found among studies could be the model used for threshold estimation.

Our results accord with results published by Mandalos et al. [9], who recommended a WS of 6 as cut-off value in order to reduce inter-observer variability. However, the authors used a very small sample size of 15 subjects, and the aim of the study was to investigate inter-observer variability and not to find a cut-off value.

Limitations of this study due to its retrospective design might be discussed. Measurements with the ORA device were taken by different examiners, and this may induce some bias. Another limitation to our study is that the included subjects are all healthy individuals; no damage in the visual fields or in the optic nerve evaluation was detected. The mean IOP in our included subjects was around 16 mmHg (16.03 mmHg with ORA and 16.67 mmHg with Goldmann). However, several subjects showed an IOP that was above 21 mmHg, introducing some bias to our results. Care should be taken when applying our findings to a wider group. Further studies are needed to evaluate BSV threshold among patients suffering from glaucoma and other ocular illnesses.

In conclusion, the Waveform Score incorporated in the ORA version 2.04 provides information on the reliability of the measurements. Two different cut-off values came from the study. If we consider the lower 95 % confidence interval for the mean as a cut-off value, a signal with a BSV lower than 7.23 should not be accepted. If we consider the reliability of the IOP measured with the ORA device for threshold estimation, a value of 6.7 BSV induced a difference of 3 mmHg in IOP measurement. When using the ORA device, we recommend that clinicians try to obtain several measurements with WS of 7 or above. Waveform Scores lower than 7 may render less reliable results.
